# Novel Mechanistic Insight into the Anticancer Activity of Cucurbitacin D against Pancreatic Cancer (Cuc D Attenuates Pancreatic Cancer)

**DOI:** 10.3390/cells9010103

**Published:** 2019-12-31

**Authors:** Mohammed Sikander, Shabnam Malik, Sheema Khan, Sonam Kumari, Neeraj Chauhan, Parvez Khan, Fathi T. Halaweish, Bhavin Chauhan, Murali M. Yallapu, Meena Jaggi, Subhash C. Chauhan

**Affiliations:** 1 Department of Pharmaceutical Sciences, University of Tennessee Health Science Center, Memphis, TN 38163, USA; Mohammed.Sikander@utrgv.edu (M.S.); Fnu.Shabnam@utrgv.edu (S.M.); Sheema.Khan@utrgv.edu (S.K.); skumari@uthsc.edu (S.K.); Neeraj.Chauhan@utrgv.edu (N.C.); Bhavinc@outlook.com (B.C.); Murali.Yallapu@utrgv.edu (M.M.Y.); 2 Department of Immunology and Microbiology, School of Medicine, University of Texas Rio Grande Valley, McAllen, TX 78504, USA; 3 Center for Interdisciplinary Research in Basic Sciences, Jamia Millia Islamia, Jamia Nagar, New Delhi 110025, India; Parvezynr@gmail.com; 4 Department of Chemistry and Biochemistry, South Dakota State University, Brookings, SD 57007, USA; Fathi.Halaweish@sdstate.edu

**Keywords:** pancreatic cancer, cucurbitacin D, mucin, miR-145 and MUC13

## Abstract

Pancreatic cancer (PanCa) is one of the leading causes of death from cancer in the United States. The current standard treatment for pancreatic cancer is gemcitabine, but its success is poor due to the emergence of drug resistance. Natural products have been widely investigated as potential candidates in cancer therapies, and cucurbitacin D (Cuc D) has shown excellent anticancer properties in various models. However, there is no report on the therapeutic effect of Cuc D in PanCa. In the present study, we investigated the effects of the Cuc D on PanCa cells in vitro and in vivo. Cuc D inhibited the viability of PanCa cells in a dose and time dependent manner, as evident by MTS assays. Furthermore, Cuc D treatment suppressed the colony formation, arrest cell cycle, and decreased the invasion and migration of PanCa cells. Notably, our findings suggest that mucin 13 (MUC13) is down-regulated upon Cuc D treatment, as demonstrated by Western blot and qPCR analyses. Furthermore, we report that the treatment with Cuc D restores miR-145 expression in PanCa cells/tissues. Cuc D treatment suppresses the proliferation of gemcitabine resistant PanCa cells and inhibits RRM1/2 expression. Treatment with Cuc D effectively inhibited the growth of xenograft tumors. Taken together, Cuc D could be utilized as a novel therapeutic agents for the treatment/sensitization of PanCa.

## 1. Introduction

Pancreatic cancer (PanCa) represents one of the most common aggressive solid malignancies and it is fourth leading cause of cancer-related deaths in the Unites States [1]. Poor prognosis, worst outcomes, and short survival are hallmarks of this cancer. Although our understanding of its etiology has increased over the past few decades, this has not resulted in a significant improvement in treatment options. Pancreatic cancer patients currently have a low survival of around eight months and ~8% five-year survival rate. PanCa is estimated to be the second leading cause of cancer deaths in the United States by 2030 [2]. Recently, gemcitabine (GEM), which is a nucleoside analog, has become an FDA-approved treatment, but, in most cases, it can only prolong survival by a few weeks [3]. Recently used combination therapies, such as FOLFIRINOX and gemcitabine/Abraxane, have shown improved efficacy, but it can cause profound toxicity [4]. Unfortunately, there are currently no curative options for advanced or metastatic PanCa [5]. The inherent resistance of PanCa to available treatment options poses a major challenge in reducing the mortality from this pernicious disease. The proper management of PanCa requires the development of incipient targeted therapies with less toxicity and a correspondingly improved efficacy.

Nowadays, natural products are of significant interest as new drug sources for cancer therapy [6]. Recently, natural bioactive agents have been shown a number of therapeutic activities including anti-cancerous properties, but inadequate bioavailability restricts their clinical use [7,8,9,10]. The cucurbitacins are oxygenated tetracyclic triterpenoids that are isolated from the Cucurbitaceae family and are well known for the acridness of edible products, like pumpkins, gourds, and squashes. They are known to exhibit potent anti-inflammatory and anti-cancer effects on various tumors [11]. Several analogues, including (B, D, E, I, F, O, P, and Q), have been shown to suppress tumor cell proliferation via the inhibition of STAT3 phosphorylation [12]. Although cucurbitacins have moderate to high toxicity, their structural properties may contribute to potential future chemotherapeutic modalities. Notably, Ding et al. (2010) observed that cucurbitacin D analogue (Cuc D) is non-toxic in peripheral blood mononuclear cells from healthy donors or in mouse primary cells [13]. Several reports have shown the anticancer activity of Cuc D in various cancer models [13,14,15]. Previously, we have shown the anticancer activity of Cuc D in cervical and prostate cancer [16,17]. Cuc D has been reported to inhibit the proliferation of doxorubicin-resistant human breast carcinoma cells [15]. In human T cell leukemia, Cuc D induce growth suppression via the inhibition of proteasome pathways [13]. Additionally, Cuc D has been shown to downregulate the heat shock protein 90 (HSP90) chaperone machinery [18]. However, the exact molecular mechanism of its action needs to be elucidated.

In the present study, we investigated whether the Cuc D could inhibit proliferation, migration, invasion, and arrest cell cycle in PanCa cells. The results of our study revealed that Cuc D significantly downregulated the Mucin 13 (MUC13) expression in PanCa in vitro and in vivo. MUC13 is aberrantly overexpressed in PanCa and the ectopic expression of MUC13 augments tumorigenic features, such as enhanced cell proliferation, cell motility, cell invasion, and in vivo tumor growth [19]. We have previously shown that miR-145 expression inversely correlates with MUC13 expression in PanCa cells and human tumor tissues [20]. These small non-coding RNAs function by selectively binding to complementary 3′ UTR regions of mRNAs, resulting in target mRNA degradation or the inhibition of mRNA translation. Our findings further report that Cuc D mediated downregulation is due to the partial restoration of miR-145 in PanCa. Additionally, Cuc D treatment suppresses the growth of gemcitabine resistant PanCa and inhibits the expression of ribonucleotide reductase (RRM1/2), key regulator proteins involved in chemo-resistance. Overall, our study is the first to suggest a potential role for Cuc D in the PanCa therapy.

## 2. Materials and Methods

### 2.1. Cell Culture

Human pancreatic cancer cell lines (AsPC-1, BxPC-3, CaPan-1, and HPAF-II) were purchased from American Type Culture Collection (Manassas, VA, USA). AsPC-1, BxPC-3, and CaPan-1 were cultured as monolayer in RPMI1640, while HPAF-II was grown in DMEM/F-12 media (HyClone Laboratories, Inc., South Logan, UT, USA). The media used were supplemented with 10% heat-inactivated FBS (Atlanta Biologicals, Atlanta, GA, USA), 1% antibiotic/antimycotic and cells were incubated at 37 °C in a humidified atmosphere (5% CO_2_ and 95% air atmosphere). DMSO was used as vehicle control throughout the study.

### 2.2. Cell Proliferation Assay

MTS assay was performed to study the anti-proliferative activity of the Cuc D as described previously [20]. Briefly, the cells were seeded in 96-well plates at a density of 2.5 × 10^3^ cells per well and allowed to stand overnight at 37 °C and 5% CO_2_ in the incubator. Next day, the cells were treated with different concentrations of Cuc D (0.1, 0.25, and 0.5 µM). DMSO was used as a vehicle control. At the time interval of 24, 48, 72 and 96 h, 25 μL of MTS reagent was added and absorbance was then taken at 490 nm after 2 h using SpectraMax plus 384 spectrophotometers, (Molecular Devices, Sunnyvale, CA, USA). The experiment was conducted in quadruplicates. The data were analyzed with respect to the vehicle control.

### 2.3. Colony Formation Assay

Colony formation assay was performed to investigate the long-term effect of Cuc D on the clonogenic potential of AsPC-1, BxPC-3, CaPan-1, and HPAF-II cells. Briefly, in a six-well plate, 500 cells were seeded per well and then allowed to grow for three days. The cells were then treated for seven days with Cuc D at different concentrations (25, 50, 100 nM). DMSO was used as vehicle control. After seven days, the media was replaced with respective growth medium for another one week. After this, the cell colonies were fixed in methanol and then stained in hematoxylin. The colonies were counted and then percentage viability was calculated with respect to the vehicle.

### 2.4. Cell Cycle Analysis

Approximately 70% confluent HPAF-II cells were treated with Cuc D (0.5 and 1 µM) for 24 h. The cells were trypsinized and then washed twice with ice cold PBS (1×). The cell pellet was resuspended in 50 μL ice cold PBS (1×) and 450 μL cold methanol and left for 1 h at 4 °C. The cells were washed twice with ice cold PBS (1×), suspended in 500 μL PBS, and then incubated with 5 μL RNase (20 μg/mL final concentration) at 37 °C for 1 h. The cells were chilled over ice for 10 min and then stained with propidium iodide (50 μg/mL final concentration for 1 h). The stained cells were further analyzed by flow cytometry (BD Accuri C6; Becton-Dickinson, Mountain View, CA, USA).

### 2.5. Migration Assay

Cell migration was carried out as per the instructions of the manufacturer in Corning’s 96-well HTS trans-well. PanCa cells (AsPC-1 and HPAF-II) were seeded at a density of 50,000 cell/well in the upper chamber of the plate containing serum-free culture medium and then treated with Cuc D for 18 h, following which cells were allowed to migrate to lower chamber containing 10% FBS. After the completion of time, the cells in the upper chamber were completely removed by cotton swab, and the cells in the lower chamber were fixed in 4% paraformaldehyde solution. These cells were further stained with crystal violet. A phase contrast microscope was used to observe the migrated cells.

### 2.6. Agarose Bead Assay

Agarose bead assay was also used to study cell migration, as previously described [19]. Briefly, the cells were mixed into a low melting point agarose solution and suspension drops were placed on plates. The cells were treated for 48 h with Cuc D and photographed while using a phase-contrast microscope.

### 2.7. Wound Healing Assay

PanCa cell migration was assessed by an in vitro wound healing assay as previously described [16]. Briefly, the cells were densely plated on a six-well tissue culture plate. A standardized wound was made while using a plastic tip of P200, followed by treatment with Cuc D (0.1 and 0.5 μM).

### 2.8. Invasion Assay

Biocoat Matrigel Invasion Chambers (BD Biosciences, San Jose, CA, USA) were used to investigate the effect of Cuc D on the invasiveness of PanCa cells as described previously [16]. The invading cells were fixed with methanol after 48 h incubation and then stained with crystal violet. The invaded cells were counted and then plotted as a percentage invasion of the cells treated with Cuc D as compared to the control.

### 2.9. Western Blotting

To determine the effect of Cuc D on expression in PanCa, Western blot analysis was performed. Protein lysates were prepared as previously described [20]. The proteins were analyzed by immunoblotting with anti-MUC13 Mab (clone PPZ020) and anti-β–Actin (Sigma, St. Louis, MO, USA).

### 2.10. Transfection and Quantitative Real-Time Polymerase

The cells were transfected using Lipofectamine 2000 (Invitrogen, Life Technologies, Grand Island, NY, USA) as per the manufacture’s protocol. Briefly, total RNA was isolated from control and Cuc D treated PanCa cells while using TRIzol™ reagent (Invitrogen, Life Technologies, Grand Island, NY, USA). 100 ng total RNA was reverse transcribed into cDNA while using specific primers that were designed for miRNA analysis (Applied Biosystems, Foster City, CA, USA) using High Capacity cDNA Reverse Transcription kit (Applied Biosystems, Foster City, CA). The mRNA expression of target genes (*MUC13*, *RRM1* and *RRM2*) was analyzed by real-time PCR and semi-quantitative PCR was performed to assess the MUC13 mRNA level by using standard three step procedure as previously described [20]. The expression of *MUC13* was normalized to *GAPDH*. The expression of miR-145 was normalized with the expression of control U6B small nuclear RNA (*RNU6B*).

### 2.11. Molecular Docking

Molecular docking was carried out while using Autodock Vina and AutoDock 4 package, as described previously [21,22]. Autodock Vina uses an advanced docking algorithm and scoring function of protein ligand interactions. As there is no MUC13 structure related information available, the selected sequence of MUC13 was modelled to generate the three-dimensional model while using I-TASSER and SWISS-MODEL server [23,24]. The SWISS-MODEL methodology includes general steps of BlastP for selected MUC13 sequence against Protein Data Base to find a suitable template. Transmembrane protease from *Mus musculus* (PDBID 2E7V) was selected as the best suitable templates and using these templates generated the homology model of MUC13. The best conformation of modelled MUC13, as predicted by SWISS-MODEL, was further validated using Ramachandran plot [25]. The conformation of the predicted model was calculated by analyzing the phi (Φ) and psi (Ψ) torsion angles while using MolProbity online server. Prior to docking analysis, the structure was amended by take out the ligand and co-crystallized water molecules, followed by the addition of polar hydrogens and Gasteiger charges while using Auto Dock Tool (ADT). The two-dimensional (2D) and three-dimensional (3D) structures of Cuc D were generated and energy as minimized while using ChemBio3D Ultra 12.0. The protein receptor molecules and ligand were converted to the appropriate docking format through PyRx. Subsequent to the preparation of coordinate files, the ligand was docked by defining a grid box around the protein active site and bound conformations, binding affinity, and possible protein-ligand interactions were studied. PyMOL viewer (Schrödinger, LLC) and “Receptor-Ligand Interactions” modules of BIOVIA/Discovery Studio 2017R2 were used for the visualization and structure analysis of the docked complexes and for generating two/three dimensional images for the analysis of hydrogen bonds and hydrophobic interactions.

### 2.12. Xenograft Study

We performed ectopic xenograft studies in mice to determine the anti-tumor effect of Cuc D. To this end, six-week old NOD-SCID gamma mice were purchased from Jackson laboratory and maintained in a pathogen-free environment. All of the procedures were carried out in accordance with the protocol that the UTHSC Institutional Animal Care and Use Committee (UTHSC-IACUC) approved. HPAF-II cells (4 × 10^6^ cells) were suspended in phosphate buffer saline (PBS) and Matrigel (BD Biosciences) solution (1:1 ratio) and subcutaneously injected on the dorsal flanks of each mouse to establish ectopic xenograft tumors in mice. Mice tumor growth was monitored while using a digital Vernier caliper. When tumor volume reached ~100 mm^3^, mice were divided into control groups and Cuc D treatment groups. The mice were treated with Cuc D (1 mg/kg bwt thrice a week; intra-peritoneally) or vehicle control (PBS). Tumor volumes were measured weekly and then calculated while using formula 0.5238 × L × W × H, where L is length, W is width, and H is tumor height. Mice were euthanized when the control mice tumor volume reached ~1000 mm^3^. The tumors were excised and processed for RNA, tissue lysates, histopathology, and preparation of slides (5μm section) at the time of sacrifice.

### 2.13. Immunohistochemistry (IHC)

IHC analysis for PCNA and MUC13 proteins was performed on formalin-fixed, paraffin-embedded xenograft tumors (5-micron sections) as described previously [20]. Briefly, the tumor tissues were deparaffinized, rehydrated, treated with 0.3 percent hydrogen peroxide, and then processed for antigen retrieval while using a heat-induced technique. The samples were processed for staining with PCNA and MUC13 antibodies after blocking with background sniper (BioCare Medical, Concord, CA, USA). The expression of these proteins was detected while using a MACH 4 Universal HRP Polymer Detection Kit (BioCare Medical) and 3,9-diaminobenzidine (DAB Substrate Kit, Vector Laboratories, Burlingame, CA, USA). The slides were counter-stained with hematoxylin, dehydrated, mounted with VectaMount (Vector Laboratories), and visualized using an Olympus BX 41 microscope (Olympus Corporation, Tokyo, Japan).

### 2.14. In Situ Hybridization

In situ hybridization technique was used in order to detect the expression of miR-145 in tissues of control and treated xenograft mice by Biochain kit (Catalog number K2191050; Biochain IsHyb In Situ hybridization kit) as described previously [20]. Briefly, the tissues were deparaffinized and then fixed in 4% paraformaldehyde in DEPC-PBS for 20 min. They were digested using 2x standard saline citrate and 0.1% Triton-X for next 25 min. The tissue was prehybridized with prehybridization solution provided with the kit for 4 h at 48 °C. This followed the hybridization of the slides with hybridization buffer and digoxigenin labelled probe (EXIQON, Woburm, MA, USA) at 45 °C overnight. The slides were blocked using 1x blocking solution that was provided with the kit after stringent washing of tissue slides with various grades of standard saline citrate. This followed the subsequent incubation of tissues overnight with the AP-conjugated anti-digoxigenin antibody. Further, the slides were washed for 5 min. with 1x Alkaline Phosphatase buffer twice.

### 2.15. Bioavailability Studies of Cuc D

Cuc D (2mg/kg) was given orally and intra-peritoneally. A 150 µL blood sample was withdrawn from retroorbital vein at 30 min., 12 h, and 48 h. 100 µL of methanol were added to the plasma samples and then mixed by vortexing. The mixture was centrifuged at 1500 rcf for 5 min. Cuc D concentration was achieved while using HPLC Dynamax liquid chromatography (Varian chromatography systems) equipped with PDA-2 (photodiode array UV detector) and Altima C-18 (Alltech analytical column, 5 µm, length 250 mm, ID 4.6 mm), which is developed with the gradient solvent system of MeOH/H_2_O and flow rate of 1 mL/min. Cuc B was used as an internal standard.

### 2.16. Statistical Analysis

Statistical analysis was performed while using an unpaired two-tailed Student *t*-test and it was employed to assess the statistical significance between the control and Cuc D-treated groups. *p* values < 0.05 were considered to be significant (* *p* < 0.05, ** *p* < 0.01, *** *p* < 0.001).

## 3. Results

### 3.1. Cuc D Treatment Suppresses Proliferation and Clonogenicity of Pancreatic Cancer Cells

**Figure 1:**
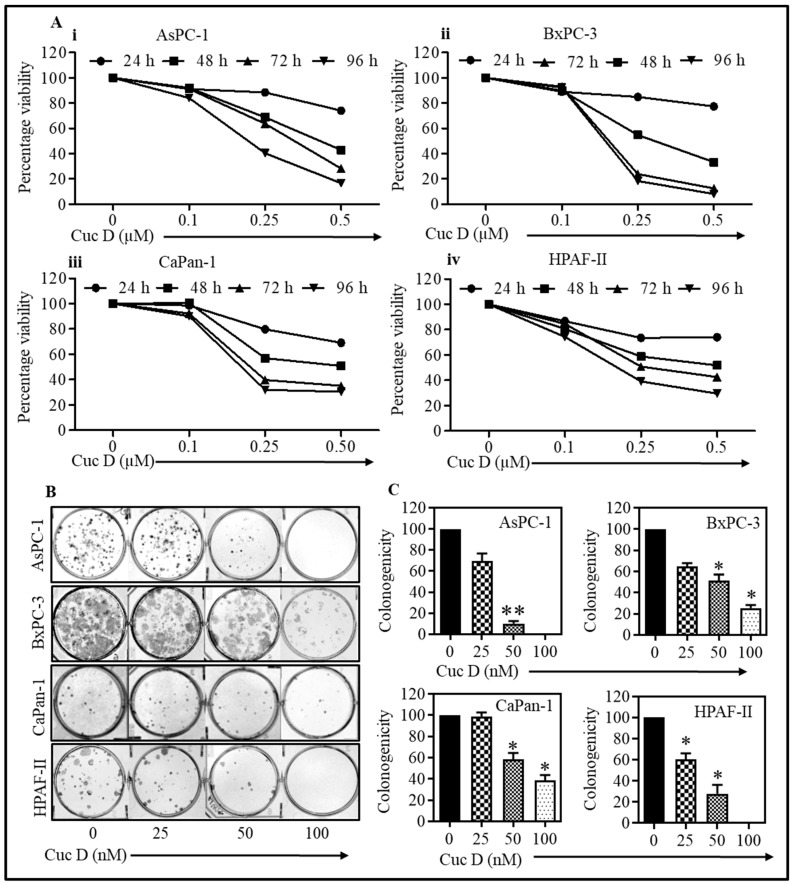
Cuc D decreases proliferation and colony formation of pancreatic cancer cells. (**A**) Cuc D decreases the proliferation of AsPC-1, BxPC-3, CaPan-1, and HPAF-II cells (**Ai**–**Aiv**). Briefly, PanCa (2500) cells were seeded in each well of 96-well plate and after overnight incubation; cells were treated with indicated concentrations of Cuc D for 24, 48, 72, and 96 h. Cell viability was assessed by MTS assay. The graph represents the percent viable cells compared to the vehicle-treated cells. Each concentration value shown in bar graph is the mean ± SE of quadruplicates wells of each group. (**B**) Cuc D inhibits colony formation ability of PanCa cells (**B**,**C**). In brief, 500 cells were seeded in each well of 6-well plates. After three days, the cells were treated with indicated concentrations of Cuc D and colonies obtained were stained with hematoxylin. Photographs were taken by UVP-gel documentation system for all cells. Bar graphs represent number of percentage of colonies formed with respect to vehicle control in each group. The experiments were performed in triplicate. Asterisk (*) denotes the significant value *p* < 0.05 when applied student’s *t*-test, and two asterisks (**) indicates as significance of p<0.01.

In this study, we investigated the anti-proliferative activity of Cuc D against pancreatic cancer cells. The anti-proliferative effects of serial concentrations of Cuc D (for 24–96 h treatment) on pancreatic cancer cells were examined while using MTS assay (Figure 1A(i–iv)). Cuc D treatment caused a dose (0.1 to 0.5 µM) and time dependent decrease in viability of AsPC-1, BxPC3, CaPan-1, and HPAF-II cells. In addition, colony formation assays were performed to assess the long-term effect of Cuc D. Treatment with Cuc D was shown to significantly inhibit colony formation when compared to the control cells in a dose-dependent manner (*p* < 0.05, Figure 1B,C). These results demonstrated that Cuc D efficiently inhibited the growth and clonogenicity of pancreatic cancer cells. The Cuc D effects were very similar in all four pancreatic cancer cell lines (AsPC-1, BxPC-3, CaPan-1, and HPAF-II) tested. Therefore, we selected AsPC-1 and HPAF-II cell lines for further studies.

### 3.2. Cuc D Treatment Effectively Arrests Cell Cycle in G2/M Phase

**Figure 2:**
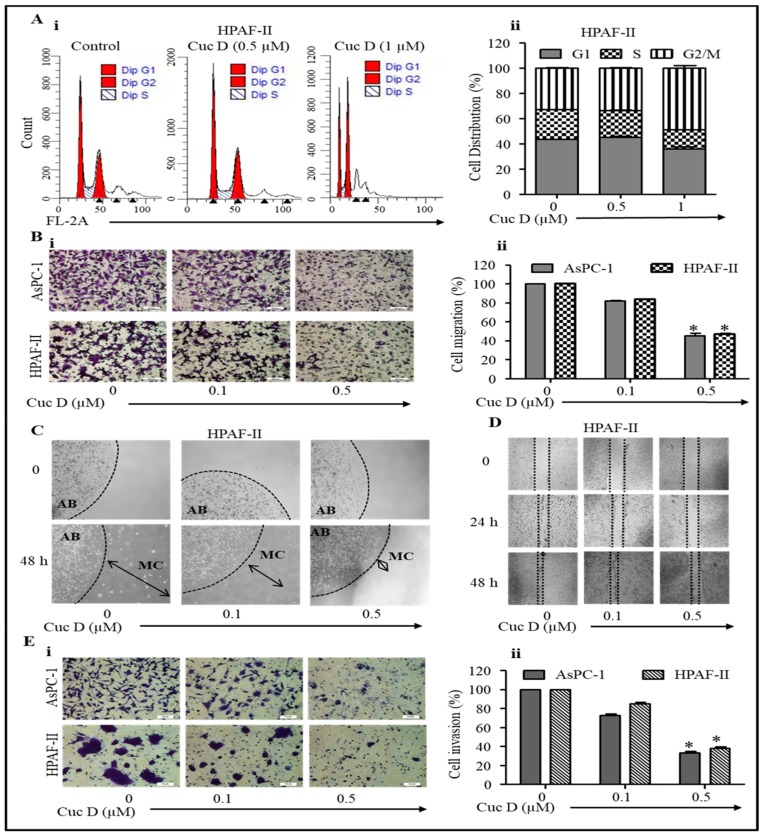
Cuc D arrest cell cycle progression, decreases migration and invasion of pancreatic cancer cells. (**A**) Effect of Cuc D on cell cycle distribution of HPAF-II cells as determined by flow cytometry. Histogram represents the cell cycle distribution in control and Cuc D treated HPAF-II cells (**Ai**,**Aii**). (**B–D**) Effect of Cuc D on cell migration of PanCa cells as determined by Boyden chamber assay (**B**)**,** agarose bead (**C**) and scratch wound (**D**). Representative images (magnification, × 20) of migratory cells in control and Cuc D treated groups at 0 and 48 h (**Bi**). Bar graph represent the quantification of migrated AsPC-1 cells of control and Cuc D treated groups (**Bii**). AB denotes agarose bead while MC denotes migratory cells (**C**). Arrows indicate the distance of PanCa cells migration in control and Cuc D treated groups (magnification, × 10). Scratch wound assay showing effect of Cuc D on HPAF-II cells (**D**). Briefly, a standardized wound was made using a 200µL micropipette tip in 80–90% confluent 12-well plate and treated with indicated concentration of Cuc D. Closure of wound was determined and photographed using phase contrast microscopy. Representative images (magnification, ×10) of migratory cells in control and Cuc D treated groups at 0, 24 and 48 h. (**E**) Effect of Cuc D on invasion of PanCa cells, as determined by a commercially available kit (BD Biosciences), as described in materials and methods. Representative images (magnification, ×20) of invaded control and Cuc D treated cells (**Ei**). Bar graph represent the quantification of invaded AsPC-1 cells of control and Cuc D treated groups (**Eii**). Student’s *t*-test was performed to analyze significant difference. Asterisk (*) denotes significant value *p* < 0.05.

Cancer progression is associated with the aberrant regulation of the cell cycle [26]. Thus, using PanCa cells, we investigated the effect of Cuc D on the cell cycle through flow cytometry. Cuc D caused a dose dependent growth arrest in the G2/M phase of the cell cycle (Figure 2A(i,ii); representative data for HPAF-II shown). The percentage of cells in the G2/M phase increased after 24 h of drug treatment as compared to the vehicle control group, as shown in Figure 2A.

### 3.3. Cuc D Treatment Inhibited Migratory Potential of PanCa Cells

The migration of epithelial cells is an important step in cancer metastasis [27]. Therefore, treatment designed to inhibit the ability of tumor cells to migrate can have a dramatic improvement in the survival of cancer patients. We therefore wanted to determine whether Cuc D could inhibit PanCa cell migratory properties through Boyden chamber assay, agarose bead assay, and wound healing assay. Treatment with Cuc D showed that fewer cells migrated through the semipermeable layer when compared to control (Figure 2B(i,ii)). The PanCa cells were trapped in the agarose beads and treated with Cuc D for 48 h, which then showed a smaller number of migratory cells from the bead compared to the control. (Figure 2C). Similar observations were also observed in a wound healing assay following Cuc D treatment, as there was a marked decrease in cell migration in the wound that was prepared by using 200 μL pipette tip (Figure 2D). Overall, these results confirm that Cuc D has an effective anti-migration capability against PanCa cells.

### 3.4. Cuc D Treatment Suppresses Invasiveness of PanCa Cells

The invasion of cancer cells is an integral process for cancer metastasis [27]. A matrigel invasion assay was used to test the effect of Cuc D on the invasive ability of pancreatic cancer cells. As shown in Figure 2E(i), Cuc D treatment significantly inhibited the invasiveness of pancreatic cancer cells starting at 0.1 μM, invaded cells were counted and then plotted as a percentage of invasion of Cuc D treated cells when compared to control (Figure 2E(ii)). These results suggest that Cuc D has potent anticancer activity for reducing the invasiveness of PanCa cells.

### 3.5. Cuc D Treatment Inhibits MUC13 Expression in PanCa Cells

**Figure 3:**
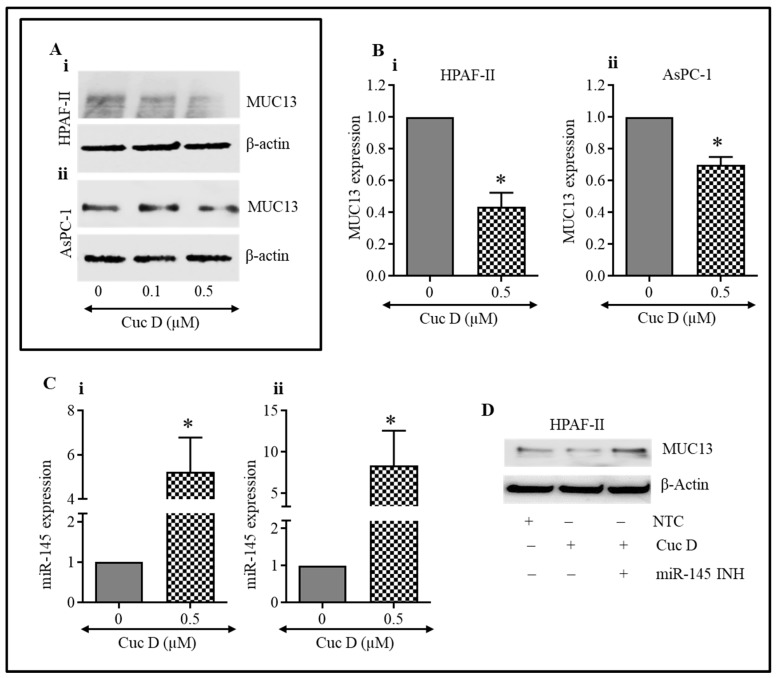
Cuc D inhibits the MUC13 expression in PanCa cells. (**A**,**B**) Effect of Cuc D on protein levels of MUC13 expression in HPAF-II and AsPC-1 cells. Effect of Cuc D on MUC13 expression in HPAF-II and AsPC-1 cells as determined by Western blotting (**Ai**,**Aii**) and qRT-PCR (**Bi**,**Bii**). Effect of Cuc D treatment on expression of miR-145 in HPAF-II (**Ci**) and AsPC-1 cells (**Cii**) as determined by qRT-PCR analysis. RNU6B was used as an internal control. (**D**) Effect of Cuc D on MUC13 expression after transfection of the cells with miR-145 inhibitor as determined by Western blot analysis. Asterisk (*) denotes significant value *p* < 0.05.

Previously, we reported the role of MUC13 as a diagnostic and therapeutic target in PanCa [19,20]. MUC13 mucin is aberrantly over-expressed in PanCa and the exogenous expression of MUC13 increases tumorigenic features, such as enhanced cell proliferation, cell motility, cell invasion, and in vivo tumor growth [19]. In the current study, we investigated the effect of Cuc D on MUC13 expression in PanCa (AsPC-1 and HPAF-II) cells. As shown in Figure 3A(i,ii), treatment with Cuc D substantially reduced MUC13 expression. We further observed that Cuc D treatment also decreases the mRNA level of *MUC13*, as evidenced by qRT-PCR analysis (*p* < 0.05) (Figure 3B(i,ii)). It was found that 0.5 μM Cuc D treatment down-regulates mRNA expression of *MUC13* (Figure 3B(i,ii)). Previously, we have shown that tumor suppressor miR-145 targets 3′ UTR of *MUC13* and, thus, downregulates MUC13 protein expression in PanCa cells [20]. MicroRNA disruption has significant implications for PanCa’s etiology, treatment, and pathogenesis. Thus, HPAF-II and AsPC-1 cells were treated with Cuc D, which increased the expression of tumor suppressor miR-145 in PanCa cells, as determined by qRT-PCR (Figure 3C(i,ii)). Furthermore, the PanCa cells were treated with Cuc D alone or in combination with miR-145 inhibitor to investigate whether Cuc D restores tumor suppressor miR-145. Herein, treatment with Cuc D decreased the levels of MUC13 and this decrease was partially reversed by the addition of miR-145 inhibitor, as demonstrated by Western blot analysis (Figure 3D). These findings collectively suggest that Cuc D significantly reduced MUC13 expression through the subsequent enhancement of miR-145 expression in PanCa.

### 3.6. Molecular Docking Analysis

**Figure 4:**
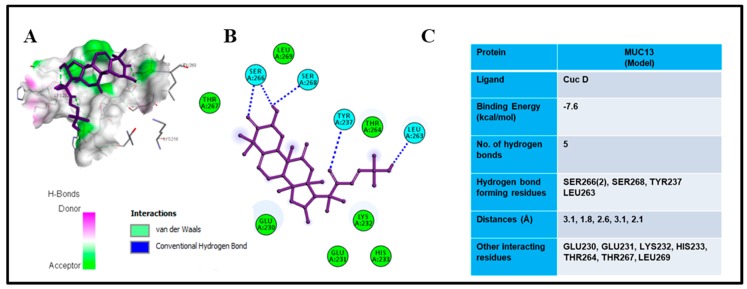
Molecular docking studies of Cuc D with MUC13. Binding view of Cuc D in the catalytic pocket of (**A**) MUC13 shows the hydrogen bond donor-acceptor residues. 2D schematic representations for the docking model of Cuc D with (**B**) MUC13. Dotted lines in different colors show various types of interactions such as hydrogen bonding, charge or polar interactions and van der Waals interactions. Table showing docking score of Cuc D with MUC13 (**C**).

Molecular docking was used to envisage existing molecular interactions of Cuc D with amino acid residues while utilizing Autodock, Autodock Vina™ combined with PyRx™ for workflow management [21,28]. In case of MUC13, the selected domain was modeled and used for docking studies. Figure 4A–C, Figures S1 and S2 present data from docking experiments. The results showed that Cuc D forms stable complexes with MUC13 and binds deep into the binding cavity of protein (Figure S1). The protein-ligand complexes were stabilized by several non-covalent interactions that are offered by the residues present in the active site cavity of predicted MUC13 model (Figure 4A,B). The results of docking revealed that Cuc D showed −7.6 kcal/mol binding energy for the MUC13 model (Figure 4C). Cuc D forms five hydrogen bonds with SER266(2), SER268, TYR237, and LEU263 of selected MUC13 model. GLU230, GLU231, LYS232, HIS233, THR264, THR267, and LEU269 are the other interacting residues (Figure 4B,C). These results suggested that Cuc D possesses high binding affinity and forms stable complexes with MUC13. Re-docking the co-crystalized inhibitors of protein into the active site cavity assessed the reliability of the applied docking protocol.

### 3.7. Cuc D Treatment Induced Gemcitabine Sensitivity in Gemcitabine Resistant PanCa Cells

**Figure 5:**
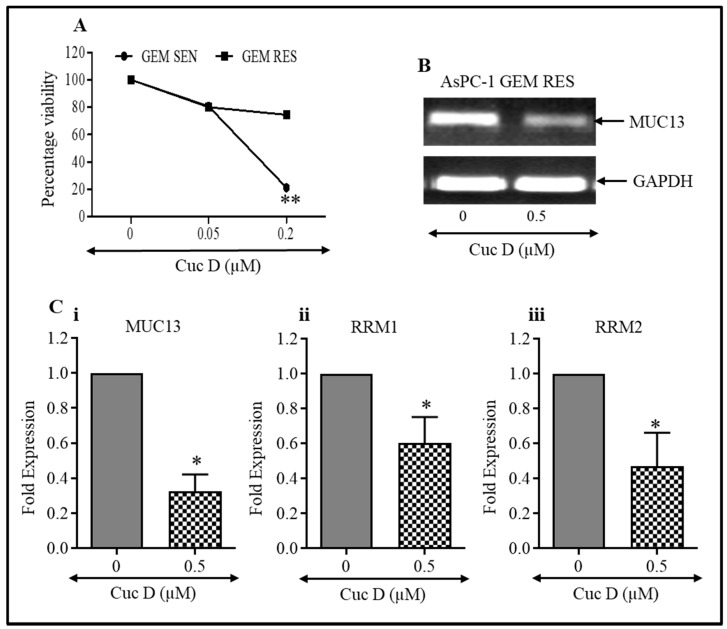
Effect of Cuc D on gemcitabine resistant AsPC-1 cells. (**A**) Briefly, 5000 cells were seeded in 96 well plate. Cells were treated with Cuc D at indicated concentrations for 48 h. Cell proliferation was determined by MTS assay. Line graph indicates the percent viability of PanCa cells with respect to vehicle control. Experiment was performed in triplicate. (**B**) Effect of Cuc D treatment on MUC13 expression in gemcitabine resistant AsPC-1 cells as analyzed by semi quantitative PCR. (**C**) Cuc D treatment decreases the mRNA expressions of *MUC13* and drug resistant genes (*RRM1* and *RRM2*) as determined by qRT-PCR. *GAPDH* was used as an internal control (**Ci**–**Ciii**). Student’s t-test was performed to analyze significant difference. Asterisk (*) denotes the significant value *p* < 0.05, and two asterisks (**) indicates a significant level of *p* < 0.01.

The current standard of care for metastatic pancreatic cancer is gemcitabine, but its success is limited due to the emergence of drug resistance [29]. Therefore, we tested whether Cuc D has anti-proliferative activity in gemcitabine-resistant AsPC-1 cells. Our data revealed a dose-dependent inhibition in the proliferation of these cells (Figure 5A). We also determined the expression of *MUC13* in AsPC-1 cells that are resistant to gemcitabine. The expression of *MUC13* decreases following treatment with Cuc D treatment, as shown in Figure 5B,C(i). It has been reported that ribonucleotide reductase (RRM1/2) have a direct role in the development of resistance [30]. Therefore, we observed the effect of Cuc D on the expression of resistant markers *RRM1* and *RRM2* by qRT-PCR. The expression of these markers with Cuc D treatment is significantly reduced, as shown in Figure 5C(ii,iii). The results revealed that Cuc D inhibits the proliferation of cells that are resistant to gemcitabine.

### 3.8. Cuc D Effectively Inhibits Tumor Growth in NOD SCID Gamma (NSG) Xenograft Mouse Model

**Figure 6:**
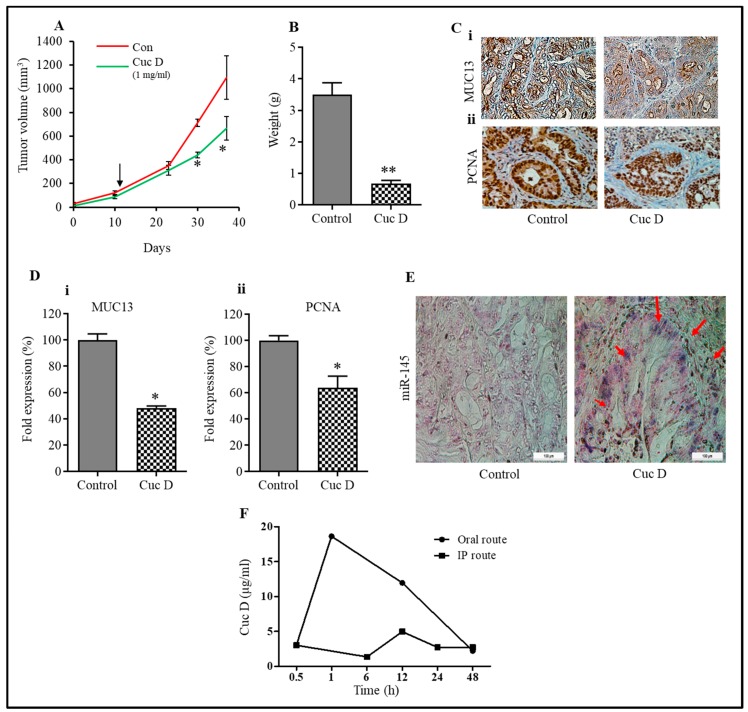
Effect of Cuc D on growth of HPAF-II cells derived xenograft mouse model. (**A**) Line graph indicates regression of HPAF-II cell-derived xenograft tumor volume in Cuc D treated mice compared to control group. Values in bar graph represent mean ± SE of six mice tumors in each group. Asterisk (*) denotes the significant value *p* < 0.05. (**B**) Tumor weight of control and Cuc D treated mice. Asterisk (**) denotes the significant value *p* < 0.01. (**C**) Effects of Cuc D on the expression of MUC13 (**Ci**) and PCNA (**Cii**) in excised xenograft tumor tissues of control and Cuc D treated mice as determined by immunohistochemistry (**D**) Representative images were captured at 20× magnification. Bar graphs represent quantification of immunohistochemistry images of MUC13 (**Di**) and PCNA (**Dii**). Student’s t-test was performed to analyze significant difference. Asterisk (*) denotes the significant value *p* < 0.05. (**E**) The effect of Cuc D on the expression of miR-145 in control and treated mice excised tumors, as determined by in situ hybridization (magnification, ×20). (**F**) Pharmacokinetics studies of Cuc D (2 mg/kg bw) in mice by oral and i.p. route.

A xenograft mouse model was developed and then used to assess the effectiveness of Cuc D to inhibit tumor growth in NSG mice. Following tumor development (~100 mm^3^), intra-peritoneal injections of Cuc D (1mg/kg body weight) and their respective vehicle control (PBS) were administered to the mice for three days/week. The volumes and weight of the tumor were measured and presented on the days that are shown in Figure 6A. At the end of the experiment, the mice were euthanized and, interestingly, Cuc D treatment showed an inhibition of tumor growth when compared to their respective control groups (Figure 6A,B). The tumor tissue samples were further analyzed for Immunohistochemistry (IHC) expression of MUC13 levels. Interestingly, in pancreatic xenograft tumors, we found that Cuc D inhibited the expression of MUC13 (Figure 6C(i)). In addition, the expression of PCNA (nuclear proliferating cell antigen) was also reduced (Figure 6C(ii)). In situ miRNA-145 hybridization (ISH) in mice tumor tissues showed that Cuc D significantly increased the expression of miRNA-145 when compared to their respective controls (Figure 6E). These in vivo data further confirm the in vitro findings that Cuc D can decrease the levels of MUC13 to attenuate cell growth (*p* < 0.05). Overall, our findings suggest that Cuc D has significant anti-tumor activity in the xenograft pancreatic tumor model.

### 3.9. Bioavailability of Cuc D in Mice

2 mg/kg body weight was given to orally and intra-peritoneally to mice to evaluate the bioavailability of Cuc D in mice. We observed that single dose administration of Cuc D (2 mg/kg body weight orally) in NSG mice showed more serum concentration as compared to the i.p. administered group (Figure 6F). Furthermore, no toxicity was observed at this dose.

## 4. Discussion

The PanCa management remains a major oncological challenge, as evidenced by the unchanged overall survival [31]. Numerous emboldening approaches have been developed in targeted therapies against PanCa, but the results in clinical tribulations are not encouraging. These limitations can overcome by using complementary and alternative medicine approaches. Natural products that are derived from plants have attracted wide attention because they are less toxic [32]. Several studies have shown that Cuc D exhibit anticancer activity via multiple pathways in various cancer models [13,14,15,16,17,33,34,35,36,37,38]. Song et al. (2013) have shown that Cuc D modulates the immune response via the activation of inflammasome [35]. Interestingly, Ding et al. (2010) observed that Cuc D did not show any toxicity in human peripheral blood lymphocytes form healthy donors [13]. There is no report of therapeutic effect of Cuc D in PanCa, to the best of our knowledge. Therefore, in the present study, we evaluated the anti-tumor effects of Cuc D in PanCa and attempted to investigate the underlying mechanisms.

**Figure 7:**
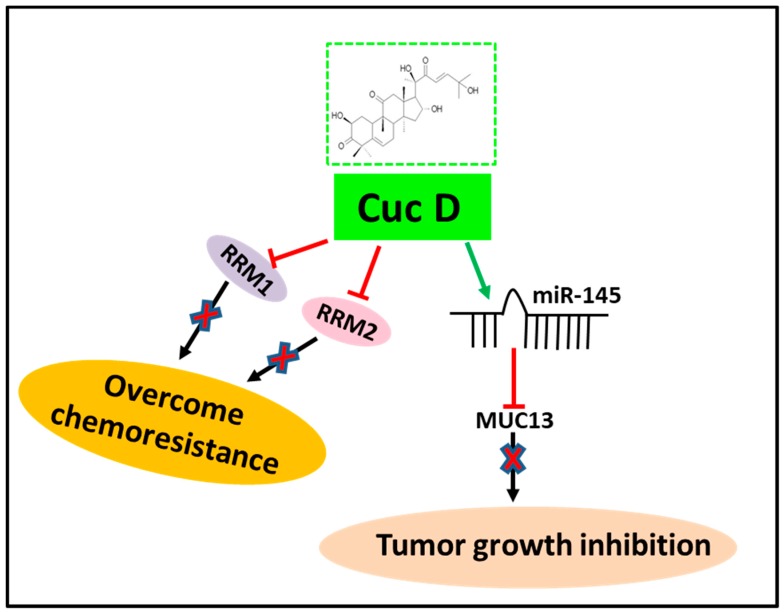
Schematic representation showing mechanism of action of Cuc D in pancreatic cancer.

Our results show that Cuc D treatment inhibited the cell growth, suppressed the colony formation, arrest cell cycle, and decreased the invasion and migration in PanCa cells. The schematic Figure 7 shows the overall effect of Cuc D on PanCa. Cuc D demonstrated a highly potent cytotoxic activity in the nanomolar range against a panel of human PanCa cells (AsPC-1, BxPC-3, CaPan-1, and HPAF-II). In line with our findings, Cuc D reportedly induced cell death in human T cell leukemia [14], breast carcinoma [15], human endometrial and ovarian cancer cells [33], and cervical cancer [16].

We observed that Cuc D mediates the growth inhibitory effect on PanCa cells *via* arresting cells in the cell cycle phase G2/M. G2 checkpoint has emerged as a captivating therapeutic target for cancer therapy. These results are consistent with the other studies, where Cuc D induce cell cycle inhibition in breast [15] and prostate [17] cancer cells. The importance of cell motility in tumor invasion makes this an attractive target for therapeutics to inhibit metastatic PanCa. Our functional experiments show that non-toxic Cuc D doses significantly decreased the PanCa cells migration and invasiveness. Previously, we have shown that Cuc D effectively suppresses the invasiveness in cervical and prostate cancer [16,17]. These findings suggest that Cuc D might be used to inhibit the metastasis of PanCa cells. Interestingly, we found that treatment with Cuc D reduces the proliferation of PanCa cells that are resistant to gemcitabine. Similarly, Cuc D treatment has been shown to overcome chemoresistance in breast cancer cells [15].

Furthermore, the downregulation of MUC13 was one of the most important findings of our studies. Mucins are identified as potential contributors to inflammation and cancer, hence they are considered to be attractive tumor markers and they are attractive targets for therapy [39,40,41]. Previously, our group has shown that MUC13 is highly upregulated in PanCa and a complex interplay of several signaling pathways regulates its expression [19]. Continuing clinical studies draw attention for the promise of mucins as therapeutic goals [42,43]. Several reports have shown the application of MUC1, MUC2, MUC4, MUC5AC, and MUC16 for mucin based therapeutic approaches [42,43]. MUC13 targeting and down-regulation could be an important approach to novel drug therapies. We for the first time provide evidence demonstrating that Cuc D can modulate mucin expression. This is in accordance with other studies, where natural bioactive agents have shown to suppress PanCa growth and down-regulate the mucin expression [44,45,46]. We further observed that the treatment with Cuc D restored miR-145 expression, as seen by qPCR analysis in both HPAF-II and AsPC-1 cells. We also noted that co-treatment with Cuc D and miR-145 inhibitor up-regulates the expression of MUC13, as determined by Western blotting. These results revealed that Cuc D mediates the inhibition of MUC13 in pancreatic cancer cells through miR-145 restoration. Nonetheless, further studies are required for evaluating these basic parameters for gaining insight of Cuc D clinical benefits.

Tumor xenograft study showed that pancreatic cancer cells that were derived from xenograft tumors in mice were inhibited by the administration of Cuc D (1 mg/kg body weight). No apparent toxicity was observed in any of these mice. These results are consistent with other cucurbitacin analogs, which also demonstrated the powerful anti- tumor activity in other cancers. Cuc D administration showed a significant reduction (*p* < 0.05) in the expression of PCNA and MUC13 protein levels in excised xenograft tumor tissues, further indicating that Cuc D has the potential to inhibit xenograft-derived human pancreatic cancer cells. In situ hybridization analysis has shown that Cuc D has been effective in restoring miR-145 expression. Overall, Cuc D has had a very promising effect on the growth of pancreatic cancer and it could be a successful therapeutic strategy for PanCa.

## Data Availability

The data that support the findings of this study are available from the corresponding author upon reasonable request.

## Supplementary Materials

The following are available online at https://www.mdpi.com/2073-4409/9/1/103/s1, Figure S1: Binding orientation of Cuc D to MUC13; Figure S2: Modeling of selected MUC13 domain structure.
