# Supplementary material for: Novel Mechanistic Insight into the Anticancer Activity of Cucurbitacin D against Pancreatic Cancer (Cuc D Attenuates Pancreatic Cancer)
Source: Cells. 2019 Dec 31;9(1):103. doi: 10.3390/cells9010103 (PMC7017063; doi:10.3390/cells9010103)
Supplement: Supplementary file 1 [file cells-09-00103-s001.zip › cells-626356-supplementary.pptx]

## Slide 1
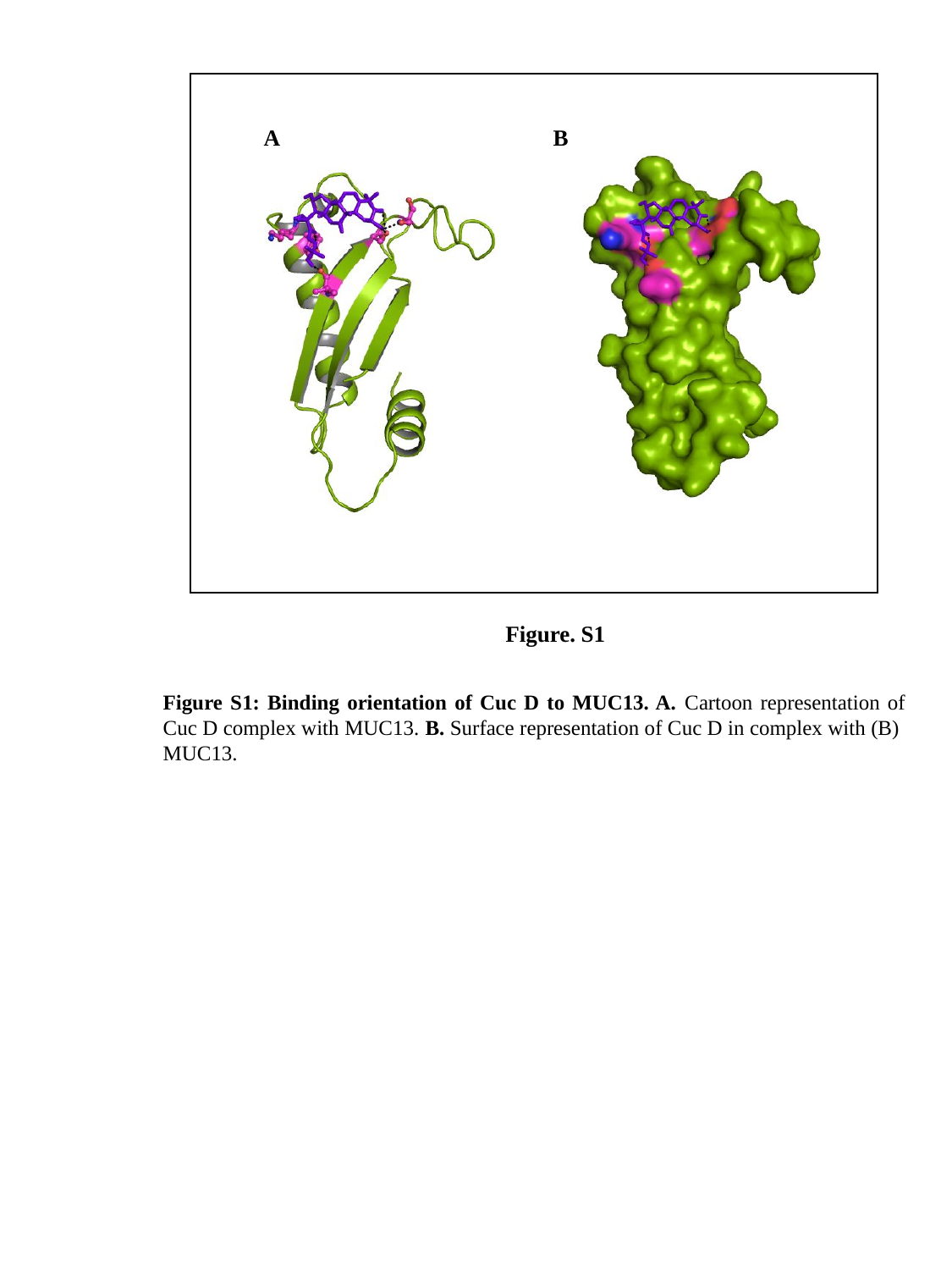

A
B
Figure. S1
Figure S1: Binding orientation of Cuc D to MUC13. A. Cartoon representation of Cuc D complex with MUC13. B. Surface representation of Cuc D in complex with (B) MUC13.

## Slide 2
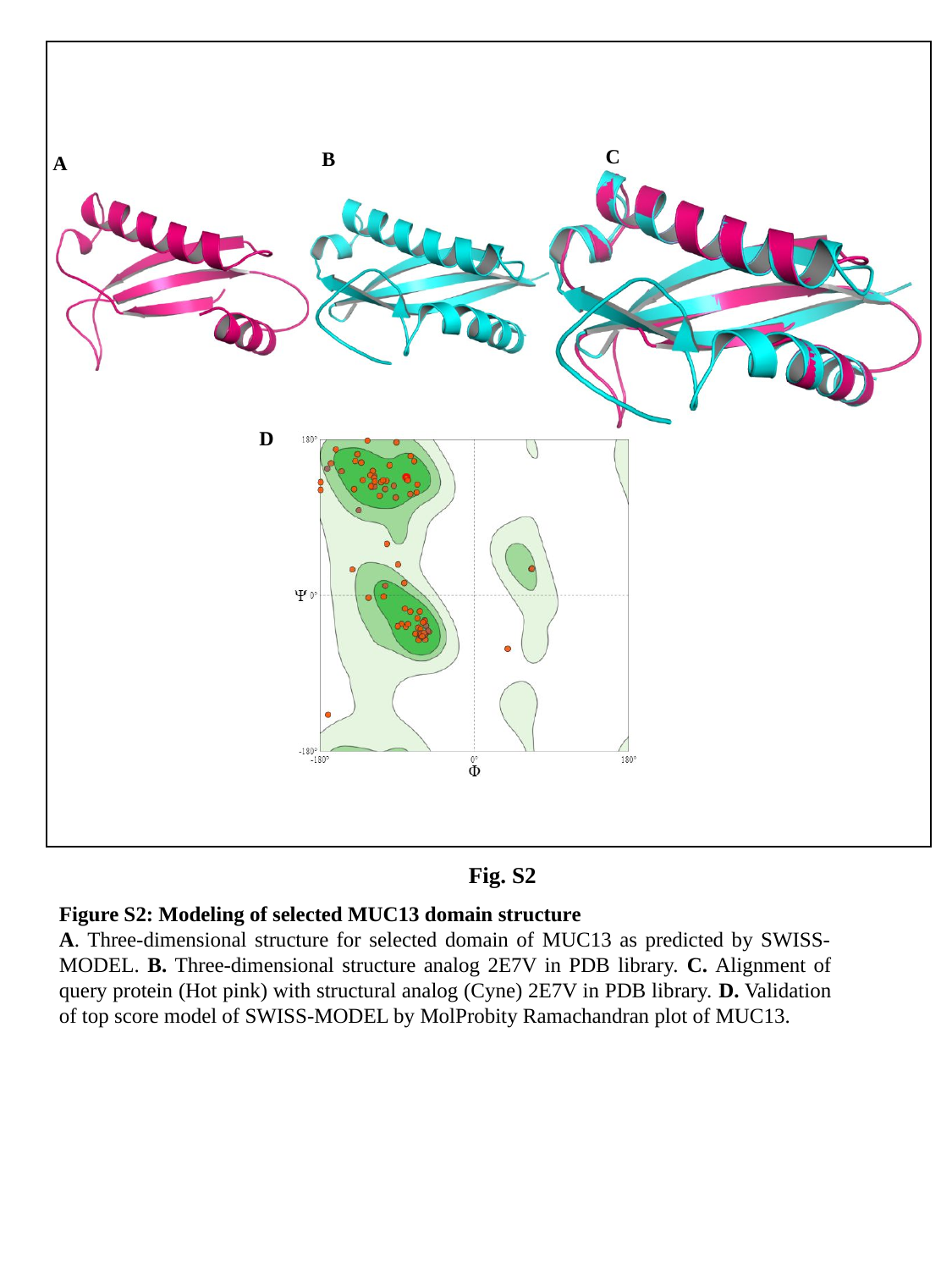

C
B
A
D
Fig. S2
Figure S2: Modeling of selected MUC13 domain structure
A. Three-dimensional structure for selected domain of MUC13 as predicted by SWISS-MODEL. B. Three-dimensional structure analog 2E7V in PDB library. C. Alignment of query protein (Hot pink) with structural analog (Cyne) 2E7V in PDB library. D. Validation of top score model of SWISS-MODEL by MolProbity Ramachandran plot of MUC13.
